# Reply to: The overwhelming role of ballistic photons in ultrasonically guided light through tissue

**DOI:** 10.1038/s41467-022-29095-w

**Published:** 2022-04-06

**Authors:** Maysamreza Chamanzar, Matteo Giuseppe Scopelliti, Adithya Pediredla, Hengji Huang, Srinivasa G. Narasimhan, Ioannis Gkioulekas, Mohammad-Reza Alam, Michel M. Maharbiz

**Affiliations:** 1 Electrical and Computer Engineering Department, Carnegie Mellon University, Pittsburgh, PA USA; 2 Robotics Institute, Carnegie Mellon University, Pittsburgh, PA USA; 3 Mechanical Engineering Department, University of California, Berkeley, CA USA; 4 Electrical Engineering and Computer Science Department, University of California, Berkeley, CA USA; 5 Chan Zuckerberg Biohub, San Francisco, CA USA

**Keywords:** Optical techniques, Imaging techniques, Microscopy, Optical imaging

**replying to** Eitan Edrei et al. *Nature Communications* 10.1038/s41467-022-29157-z (2022)

In a previous publication^1^, we introduced the concept of virtual optical waveguides capable of guiding and confining light without physically inserting optical components into the medium. We showed that ultrasound can locally change the refractive index in transparent and scattering media to sculpt in situ gradient-index (GRIN) optical waveguides. These waveguides can therefore be formed where external waveguides cannot be placed non-invasively. Follow-up work has also demonstrated the utility of this technique for confining light in tissue^2,3^.

Edrei and Scarcelli^4^ have tried to understand the underlying mechanisms of ultrasonic light guiding. Their letter acknowledges that virtual waveguides can guide scattered photons, and subsequently focuses on clarifying the relative effect of guiding ballistic versus scattered photons. Unfortunately, their letter draws incorrect and overly broad conclusions about the potentials and limitations of ultrasonic optical confinement based on very narrow assumptions and analysis of one specific example, which is very different from the virtual waveguides presented in our original publication^1^. Here, we address the technical aspects of their claims, and also present a fundamental response to their observation.

We structure our response into three parts:

As an initial matter, the analysis and conclusions of Edrei and Scarcelli^4^ are based on simulating a specific narrow step-index waveguide. This is very different from the GRIN waveguides presented in our paper^1^. Using simulations of the correct waveguide type and dimensions, we show that this discrepancy is significant, as the two different types of waveguides result in very different performances. Therefore, the quantitative characterizations and the associated conclusions by Edrei and Scarcelli^4^ do not apply to the ultrasonic sculpting technique in our paper^1^, even when using the performance metrics that they have used. Second, the comparison metrics used by Edrei and Scarcelli^4^ are based on arbitrary thresholds and lead to errors. Importantly, these metrics cannot properly evaluate the confinement of our GRIN waveguides^1^. Here, we present proper metrics that characterize light throughput enhancement and photon distribution. Lastly, the conclusions by Edrei and Scarcelli^4^ are fundamentally based on limiting assumptions about how the ultrasonically sculpted optical waveguides can be used in comparison to an external lens. We show that in addition to guiding scattered photons, the virtual nature of these waveguides provides unique geometrical advantages when properly used, even if only ballistic photons are considered.

## Waveguide type and size

Edrei and Scarcelli^4^ simulate a very narrow (core radius = 0.1 mm) step-index waveguide. This is different from the virtual waveguide in our paper in two important ways: First, our waveguide has a gradient-index and not a step-index profile. Second, our waveguide is much wider (core radius = 0.888 mm). Regarding the former, Edrei and Scarcelli^4^ use the TracePro software, which cannot simulate GRIN waveguides inside scattering media, a fact confirmed by the developers of TracePro. They claim “for a multimode scenario and a similar NA, the difference in index profile is not expected to affect scattering properties”^4^. This is not correct.

**Fig. 1:**
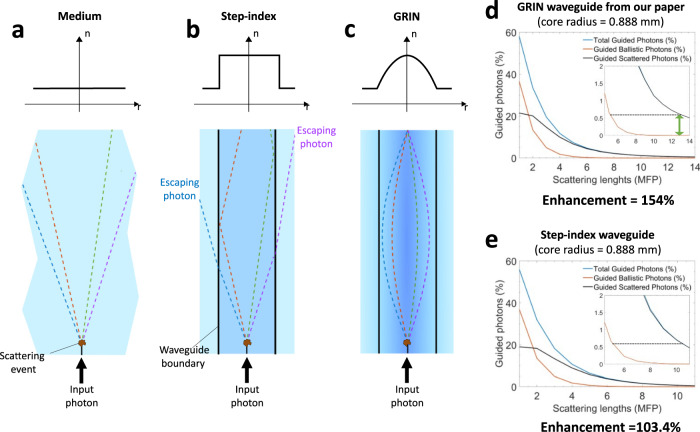
Difference in guiding scattered photons between step-index and GRIN waveguides. Schematic showing optical ray tracing after a scattering event in (**a**) a simple medium, (**b**) a step-index waveguide, and (**c**) a GRIN waveguide. When a photon undergoes a scattering event in the medium, it is deflected and dispersed. If the same scattering event happens within a step-index waveguide core (Δn = 0.002), the scattered photon will travel to the waveguide core-cladding interface, where it can either escape the waveguide or deflect back into the core by total internal reflection, depending on the scattering angle. In a GRIN waveguide with (Δn = 0.002), scattered photons are continuously refracted and rerouted towards the target location. **d** Percentage of scattered, ballistic, and total photons at different optical depths for a virtual GRIN waveguide (Ultrasound ON) with Δn = 0.002. The penetration improvement at 0.6% caused by guided scattered photons is 154%. The green arrow shows the difference between total guided photons and guided ballistic photons. **e** When the same parameters are used for a step-index waveguide, the penetration improvement is reduced to 103.4%.

Light is guided in a step-index waveguide through total internal reflections at the boundary between the core and cladding, whereas GRIN waveguides guide light by gradually refracting photons everywhere in the waveguide with a gradient refractive index profile. The two waveguide types effectively guide ballistic photons similarly (with small differences in modal and spectral dispersion). However, when it comes to guiding scattered photons, the two waveguide types are fundamentally different. To understand the mechanism, let’s consider the simple case of a photon that undergoes a scattering event in the medium. Depending on the scattering angle, this photon will be deflected away from the target location, as shown in Fig. 1a. If this scattering event happens inside the step-index waveguide core, the photon’s trajectory cannot be corrected until it reaches the core-cladding boundary, and it may scatter multiple times along the way and escape the waveguide core without experiencing total internal reflection (Fig. 1b). In contrast, the photon’s path is subject to a corrective guiding mechanism immediately after the scattering event in the GRIN waveguide, because photons are continuously refracted everywhere in the waveguide (Fig. 1c). In addition, the GRIN waveguide can confine and guide scattered photons that would be otherwise lost in the step-index waveguide. Consequently, GRIN waveguides are more effective in guiding scattered photons than step-index waveguides.

To quantify the discrepancy, first, we used a physically-accurate Monte Carlo renderer that we have recently developed^5^ to simulate the virtual GRIN waveguide with a core radius of 0.888 mm (used in our paper^1^) with the same Δn = 0.002, which is also used in ref. ^4^. As shown in Fig. 1d, this GRIN waveguide that has a core radius matching the one in our original paper provides much higher depth enhancement compared to the narrow step-index waveguide reported by Edrei and Scarcelli^4^ (154% versus 20%). Therefore, even using the same performance metric of Edrei and Scarcelli^4^, the discrepancy between the performance of their waveguide and what we had presented in our paper^1^ is significant.

In the next step, we simulated a waveguide with the same core radius of 0.888 mm, but with a step-index profile (Fig. 1e). We used the TracePro software that was employed by Edrei and Scarcelli^4^. In this case, the improvement of penetration depth is 103.4%, significantly lower than what the GRIN waveguide can provide, i.e., 154%. Therefore, contrary to what Edrei and Scarcelli^4^ claim, the refractive index profile indeed matters.

Additionally, when comparing this step-index waveguide (0.888 mm core radius) with the one used by Edrei and Scarcelli^4^ (0.1 mm core radius), we can see that the choice of a very narrow waveguide in their simulations significantly contributes to the observed depth enhancement, i.e., 30% versus 103.4% for the larger core waveguide that matches what we had in our paper^1^. The very selective choice of parameters for “success” in their simulations leads to invalid conclusions about the virtual waveguides in our paper.

It is also worth noting that while Edrei and Scarcelli^4^ acknowledge the anisotropy factor averages around *g* ~ 0.9 in most biological tissues, they have used *g* = 0.85 in their simulations, which is smaller than the smallest value in the cited references. Nevertheless, they show the penetration depth is only reduced by 10%, from 30% for *g* = 0.9 to 20% for *g* = 0.85. It can be seen that the effect of the anisotropy factor, in this case, is much smaller than the contribution of the refractive index profile and the waveguide dimensions (see Table 1). Therefore, their claim that the effectiveness of our method “strongly depends on light being scattered predominantly in the forward direction...” is not substantiated.

**Table 1:** Enhancement of penetration depth for different virtual waveguides.

Waveguide size	Core radius = 0.888 mm (matching our paper^1^)		Core radius = 0.1 mm (reported by Edrei and Scarcelli^4^)	
Waveguide type	GRIN	Step-index	Step-index (*g* = 0.9)	Step-index (*g* = 0.85)
Enhancement	154%	103%	30%	20%

Additionally, Edrei and Scarcelli^4^ conclude: “…the addition of guided scattered photons is expected to be minor in terms of total flux arriving at the desired location.*”* It is not clear how they have arrived at this conclusion. Even their step-index waveguide analysis (Fig. 1b in ref. ^4^) shows that at the threshold of 0.6%, guided scattered photons constitute more than 50% of the total flux arriving at the target location. Our simulations of the GRIN waveguide show that at the same threshold, more than 99.97% of the total arriving flux is composed of guided scattered photons (vertical green arrow in Fig. 1d inset). Moreover, at the 20% threshold, where 20% of the input photons is guided, half of the photon flux consists of guided scattered photons.

## Metrics and comparison

Even using the same metrics as in their letter, Edrei and Scarcelli^4^ judge the performance of our ultrasonic GRIN waveguides incorrectly (Table 1). Moreover, the chosen metrics^4^ (e.g., the *arbitrary* threshold for fluorescent emission and the absolute percentage of photons) are not the best to quantify the performance. For example, the reported enhancement in ref. ^4^ depends on the threshold, which is arbitrarily chosen to be 0.6%. Here, we suggest appropriate metrics to evaluate the improvements.

**Fig. 2:**
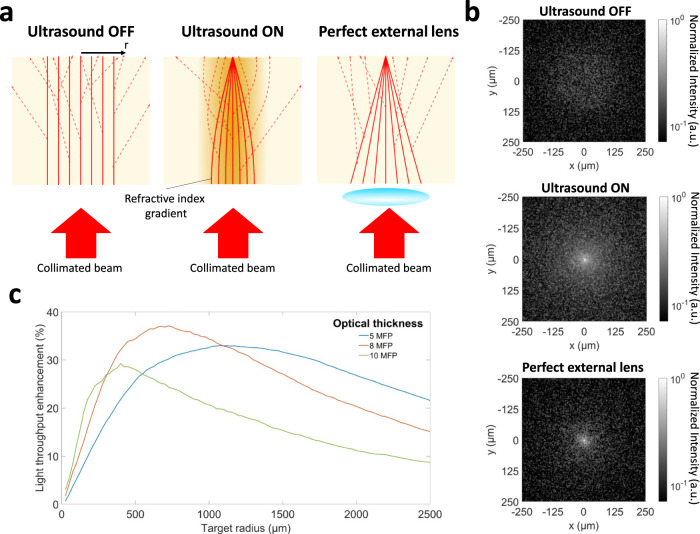
Comparison of the spatial distribution of guided photons between a virtual GRIN waveguide and a perfect lens. **a** Schematic illustration of a collimated beam of light (ultrasound OFF) through a scattering medium, a virtual GRIN waveguide (Ultrasound ON), and an external lens confining light through the medium. The target radius is shown as r on the schematic. **b** Images of scattered photons at the optical depth of 6 MFP for three cases of Ultrasound OFF, virtual waveguide (Ultrasound ON), and the external lens. **c** Light throughput enhancement for the virtual waveguide (Ultrasound ON) relative to a perfect external lens with matching focal spot size.

The characterization of improvements would not be meaningful without considering the spatial distribution of guided photons as well as a relative comparison with a baseline scenario. Figure 2a illustrates how light is confined in a scattering medium by the virtual waveguide (ultrasound ON), in comparison with two baselines: the case of no ultrasound (ultrasound OFF) and the case of a perfect aberration-free external lens. The images of scattered photons at a depth of 6 MFP for these three cases are shown in Fig. 2b. The spot sizes of the external lens and the virtual waveguide match (5 µm). In these simulations, we assumed a perfect external lens with no transmission attenuation. Therefore, the power reaching the sample surface is the same for all cases.

It is important to note that because the virtual GRIN waveguide is formed inside the scattering volume (unlike the external lens), it guides both ballistic and scattered photons towards the focal point. Naturally, this confinement is imperfect for scattered photons: due to the random scattering angles, not all scattered photons will arrive exactly at the focal point; instead, some of them end up in areas around the focal point. Figure 2b shows that the virtual GRIN waveguide confines a larger number of scattered photons around the focal point compared to the external lens. This relative improvement decreases by increasing the radial distance from the focal point. Depending on the application, it might be desirable to increase the photon flux that reaches different areas around the focal point. For example, when delivering light to a tumor, a larger target area might be desired compared to when the intended target is a single cell. With this in mind, Fig. 2c shows the relative enhancement of total light throughput (ballistic and scattered) using the virtual waveguide compared to the external lens as a function of the radius of the target area. At each optical depth, the relative light throughput enhancement increases as a function of radius until it reaches a maximum, and then decreases. For example, at a depth of 8 MFP, light throughput can be significantly enhanced by up to 37% using the virtual waveguide compared to the perfect external lens. The light throughput enhancement and spatial distribution of confined photons can be optimized for any specific application by changing the virtual waveguide parameters. The effects of different parameters of the virtual waveguide on the light throughput enhancement and the optimization strategy are discussed in a recent work^6^, where we have shown that significant light throughput enhancement in highly scattering media, including biological tissue, is possible using virtual optical waveguides sculpted by ultrasound.

## Use-case in comparison with an external lens

In their letter, Edrei and Scarcelli^4^ show that an external lens can focus light into a scattering medium and conclude that “light delivery into tissue is not dramatically enhanced by ultrasonic guiding in practical scenarios.” Here, the underlying premise is that the virtual waveguides can only be used for light delivery *from outside* into the medium, similar to an external lens. However, this premise is inaccurate. For example, a virtual waveguide can be formed using ultrasound deep into the medium, where an external physical lens cannot be placed non-invasively. Additionally, it is not necessary to use virtual waveguides only as replacements for lenses. On the contrary, as we have recently shown, lenses and virtual waveguides can be used in tandem to extend the reach and flexibility of external optics^7^.

Even if we accept the premise of Edrei and Scarcelli^4^, our simulations above demonstrate that light throughput can still be significantly enhanced compared to an external lens due to the guiding of scattered photons by the virtual waveguides.

It is also worth highlighting that light coupling into the virtual waveguides needs to be optimized to achieve the best guiding performance; this was explicitly mentioned in our original paper^1^: “…the input beam can be optimized to match the NA and maximize the coupling efficiency.” As shown experimentally in the Supplementary Material section, by choosing the proper input light coupling conditions, light confined by the virtual waveguide results in a much higher contrast ratio of 7.29 ∓ 0.16 compared to the contrast ratio of 2.81 ∓ 0.07 achieved using an external aspheric lens (Supplementary Fig. 1c, d).

Overall, the improvements of virtual ultrasonic optical waveguides depend on the parameters of the waveguide such as frequency, amplitude, and pattern of ultrasound, waveguide dimensions, input light conditions as well as the properties of the medium. Our simulation and experimental examples show that these parameters can be optimized to achieve very large enhancements, and this is a fertile area for future investigations.

## Conclusion

The simulations and experiments in this response letter prove that the argument by Edrei and Scarcelli^4^ that the ultrasonic guiding of light *“*…overwhelmingly affects ballistic photons, and not scattered photons, thus the improvement compared to current optical modalities is marginal at best*”* is not correct. This is because: (i) the virtual waveguides significantly enhance the confinement of scattered photons, when comparing light throughput of the waveguide with an external lens having the same spot size (Fig. 2c); and (ii) the virtual waveguides provide unprecedented geometrical advantages, as demonstrated by comparing the contrast achieved with the waveguide and the external lens using the same input beam (Supplementary Fig. 1d).

We conclude by pointing out that the concept of virtual waveguides in our original paper^1^ arose from our motivation to devise a technique that created virtual waveguides non-invasively within the medium, not to design a method that would replace a conventional lens; what would be the point? Our excitement was, and continues to be, that we can form these waveguides deep in the target medium where external optical components cannot be inserted non-invasively, something that opens up a plethora of new possibilities, especially when this technique is used in tandem with conventional optics^7^. Our theoretical and experimental results suggest that there are indeed several unprecedented advantages in using this technique. Therefore, the statement “light delivery into tissue is not dramatically enhanced by ultrasonic guiding in practical scenarios” is overly strong and vague. For what scenarios? For what parameters? Using what simulation paradigms? As shown in this letter and other publications^2,3,7,8^, our presented technique can be advantageous in many specific scenarios where the general statements in the letter by Edrei and Scarcelli^4^ are simply not valid. Finally, we emphasize that the performance of these virtual waveguides is a function of many parameters that must be optimized, based on which the utility of this technique should be judged in the context of intended specific applications.

## Supplementary information

Supplementary Information

## Data Availability

The data that supports the findings of this study are available from the corresponding author, M.C., upon reasonable request.
