# Supplementary material for: Reply to: The overwhelming role of ballistic photons in ultrasonically guided light through tissue
Source: Nat Commun. 2022 Apr 6;13:1872. doi: 10.1038/s41467-022-29095-w (PMC8987077; doi:10.1038/s41467-022-29095-w)
Supplement: Supplementary file 1 — Supplementary Information [file 41467_2022_29095_MOESM1_ESM.pdf]

SUPPLEMENTARY INFORMATION

As mentioned in our original paper[1], the input coupling to the waveguide should be optimized. In our paper, the input beam diameter (> 10 mm) was much wider than the narrow (~2 mm) central refractive index peak of our GRIN waveguide[1]. Because of this mismatch, not all the light from the input beam was coupled to the central waveguide (Supplementary Fig. 1a). Instead, some light was coupled to the surrounding high index rings, and the rest contributed to a large background. Therefore, the reported extinction ratios[1] should *not* be interpreted as the best possible contrasts. The experiments in our original paper[1] were designed to demonstrate the behavior of the virtual waveguide (very novel results at the time), and were not optimized for comparison with other optical techniques in terms of contrast. Below, we demonstrate experimentally that the input coupling conditions can significantly affect the achieved contrast (Supplementary Fig. 1b).

Also, our experiments compare confinement of light through 22 mm of 0.2% intralipid solution (same concentration used in ref. 4, but much deeper), when the input beam is coupled to the central GRIN waveguide, versus focused using an external aspheric lens (Supplementary Fig. 1c,d). The lens (model #49-103, Edmund Optics) has an effective focal length of 31.25 mm, and the coating provides less than 1.5% reflection in the visible range. The optical power was kept the same on the surface of the sample for both cases to compensate for the light attenuation when passing through the lens.

This experiment was repeated multiple times (n=3, using different batches of the scattering medium). The contrast ratio for the virtual waveguide (i.e., 7.29 ∓ 0.16) is much higher than 2.81 ∓ 0.07 achieved using the external lens. This is because the virtual waveguide overcomes the tradeoff between the focal distance and spot size of a conventional lens[7]. This geometrical advantage can also be achieved in non-scattering media, where the virtual waveguide exclusively guides ballistic photons.

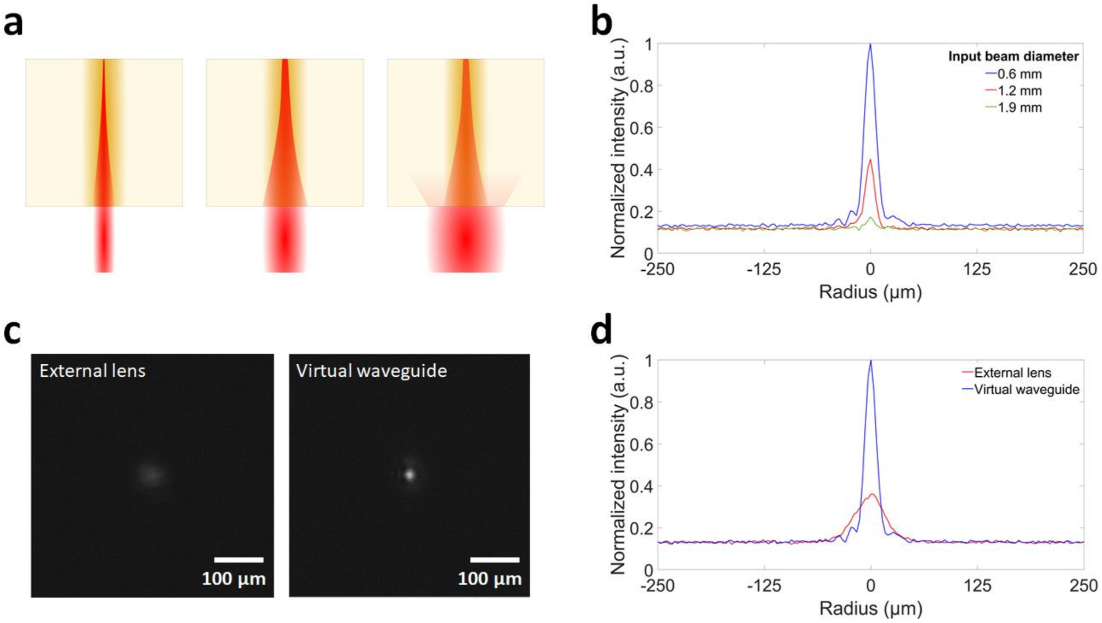

Supplementary Fig. 1. Effect of input coupling on light confinement in virtual waveguides. (a) Schematics showing the effect of the input beam diameter on light coupling to the virtual GRIN waveguides. (b) Radial cross-sections of the output beams experimentally measured in a scattering solution of IL 0.2% for three different input beam diameters. For larger input beams, the achieved contrast ratios are lower. The contrast is very large (> 7) for an input beam diameter of 0.6 mm, and it decreases to ~1.5 if light is not efficiently coupled to the waveguide for an input beam diameter of 1.9 mm. The effective waveguide width is ~1.2 mm. (c) Images of light confined through 22 mm of the scattering medium using an external lens and the virtual waveguide for an input beam diameter of 0.6 mm. (d) Radial cross-section of the beams in (c).
